# Caspase Inhibition in Select Olfactory Neurons Restores Innate Attraction Behavior in Aged *Drosophila*

**DOI:** 10.1371/journal.pgen.1004437

**Published:** 2014-06-26

**Authors:** Takahiro Chihara, Aki Kitabayashi, Michie Morimoto, Ken-ichi Takeuchi, Kaoru Masuyama, Ayako Tonoki, Ronald L. Davis, Jing W. Wang, Masayuki Miura

**Affiliations:** 1 Department of Genetics, Graduate School of Pharmaceutical Sciences, The University of Tokyo, Hongo, Bunkyo-ku, Tokyo, Japan; 2 PRESTO, Japan Science and Technology Agency (JST), Gobancho, Chiyoda-ku, Tokyo, Japan; 3 CREST, Japan Science and Technology Agency (JST), Gobancho, Chiyoda-ku, Tokyo, Japan; 4 Neurobiology Section, Division of Biological Sciences, University of California, San Diego, La Jolla, California, United States of America; 5 Department of Neuroscience, The Scripps Research Institute Florida, Jupiter, Florida, United States of America; 6 Department of Biochemistry, Graduate School of Pharmaceutical Sciences, Chiba University, Inohana, Chuo-ku, Chiba, Japan; New York University, United States of America

## Abstract

Sensory and cognitive performance decline with age. Neural dysfunction caused by nerve death in senile dementia and neurodegenerative disease has been intensively studied; however, functional changes in neural circuits during the normal aging process are not well understood. Caspases are key regulators of cell death, a hallmark of age-related neurodegeneration. Using a genetic probe for caspase-3-like activity (DEVDase activity), we have mapped age-dependent neuronal changes in the adult brain throughout the lifespan of *Drosophila*. Spatio-temporally restricted caspase activation was observed in the antennal lobe and ellipsoid body, brain structures required for olfaction and visual place memory, respectively. We also found that caspase was activated in an age-dependent manner in specific subsets of *Drosophila* olfactory receptor neurons (ORNs), Or42b and Or92a neurons. These neurons are essential for mediating innate attraction to food-related odors. Furthermore, age-induced impairments of neural transmission and attraction behavior could be reversed by specific inhibition of caspase in these ORNs, indicating that caspase activation in Or42b and Or92a neurons is responsible for altering animal behavior during normal aging.

## Author Summary

The approaching era of an “aging society” is receiving considerable attention amongst biomedical researchers in advanced nations. In order to understand the molecular mechanisms underlying age-related alterations of neural circuitry, we focused on caspase-3, a cysteine protease that induces apoptotic cell death, using the fruit fly *Drosophila melanogaster*, a model often used to study aging due to a short lifespan of approximately 30–60 days. Here, we describe the spatiotemporal activation of caspase-3 in aged fly brains and show that caspase-3 is specifically activated in select olfactory neurons essential for innate odor attraction behavior. Furthermore, we discuss how inhibition of caspase-3 activation in those select olfactory neurons can rejuvenate the sensitivity of innate attraction behavior in aged flies. These findings suggest that caspase-3 plays an active role in producing age-related alterations to neuronal physiology and circuit function associated with animal behavior.

## Introduction

Neuronal dysfunction and cell death are hallmarks of age-related neurodegenerative disorders, such as Alzheimer's disease. Epidemiological and biomedical studies have demonstrated that both genetic and age-related factors are crucial for the development and progression of these disorders. Attempts to understand the underlying mechanism of functional alterations in neural circuits during “normal aging” are receiving considerable attention [1]–[3] and should provide new insights toward preventing and treating age-related disorders. However, our knowledge about whether and how neural circuits are remodeled and/or maintained during normal aging is still very limited.

Caspases are highly conserved cysteine proteases, which function as central regulators of apoptosis [4], [5]. Knockout mice lacking *caspase-3*, *caspase-9*, or the caspase activator, *apaf-1*, all exhibit reduced neuronal apoptosis and brain malformation [6]–[11], indicating that caspases are essential for normal brain development. In addition to their role in apoptosis, non-apoptotic roles for caspases, particularly in the nervous system, are being reported *in vivo* [12]. These roles include dendritic pruning in the developing *Drosophila* [13], [14], song habituation in birds [15], [16], synaptic long-term depression (LTD) in rat hippocampal neurons [17], [18], synaptic maturation of olfactory sensory neurons in mice [19], and early synaptic dysfunction in a mouse model of Alzheimer's disease [20], [21]. Although the essential role of caspases in developing and adult brains has been documented, the *in vivo* activation pattern of caspases has not yet been systematically investigated.

In this report, we began with mapping caspase activity throughout the entire lifespan of the fruit fly. Using a genetic probe for caspase-3-like activity (DEVDase activity) [13], we revealed spatiotemporal caspase activation in the adult brain. Moreover, we found that this caspase activation was particularly prominent in the antennal lobe (AL) and ellipsoid body, which are brain structures responsible for olfaction and visual place memory, respectively [22]–[24]. Interestingly, when we further investigated caspase activity in the antennal lobe, we determined that caspases were activated in an age-dependent manner in select ORNs, particularly in Or42b and Or92a neurons that are essential for mediating innate attraction to food odors [25], and that elevation of caspase activity caused ORN death. Furthermore, two-photon calcium imaging of projecting neural dendrites (secondary neurons receiving input from ORNs) indicated that aging reduced sensitivity of the related olfactory glomeruli, which could be suppressed by the expression of p35, a caspase inhibitor. Lastly, we found that the age-related impairment of innate attraction behavior was also significantly suppressed by the inhibition of DEVDase in Or42b and Or92a neurons. Taken together, our data suggest that caspase activation in the aging brain is spatio-temporally regulated and actively contributes to age-related alterations of neural function.

## Results

### DEVDase activation is spatio-temporally regulated in the aging adult brain

**Figure 1:**
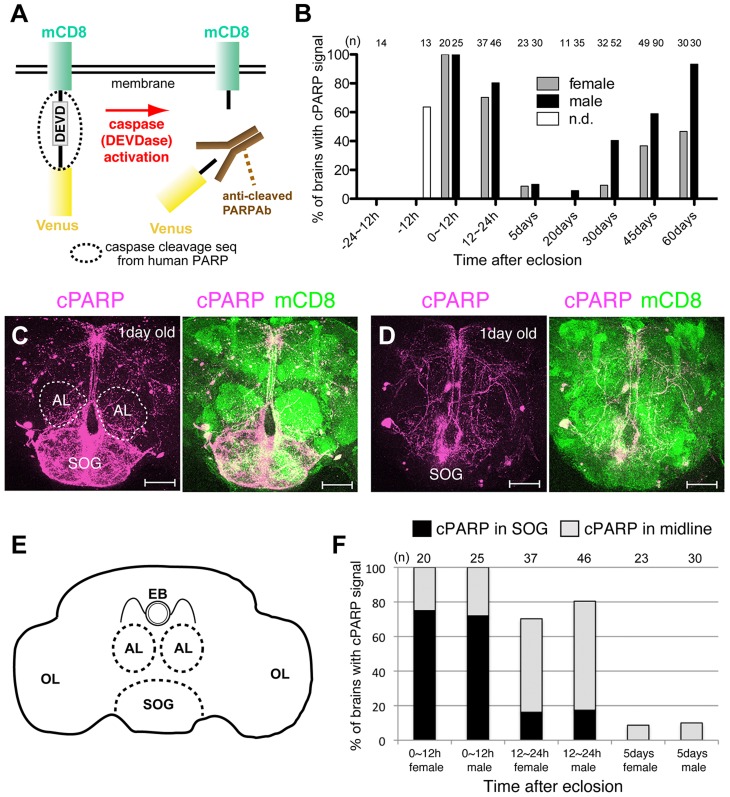
Spatio-temporal activation of DEVDase in adult *Drosophila* brains. (A) DEVDase activity detection with mCD8::PARP::Venus. Human anti-cPARP antibodies specifically recognize the N-terminal amino acid sequences of Venus that are generated by the cleavage of mCD8::PARP::Venus. (B) Percentages of brain samples with any cPARP signal at each time point are shown. “n” indicates the number of brains examined. (C, D) cPARP signals in young fly brains (1-day-old). A brain with cPARP signal near midline and the subesophageal ganglia (SOG) (C) and only near midline, without intense signals in the SOG (D). Circles of broken lines are antennal lobes (ALs). cPARP signal and mCD8::PARP::Venus expression are shown in magenta and green, respectively. Scale bar: 50 µm. (E) Schematic drawing of a *Drosophila* adult brain. The regions outlined by broken lines are ALs and SOGs. The ellipsoid body (EB) is located on the dorsal side of the AL. OL: optic lobe. (F) Graph indicating the percentage of young brains with cPARP signals. Genotypes: (B–D, F) *elav-Gal4;;UAS-mCD8::PARP::Venus.*

To monitor DEVDase activity in the brain of the adult *Drosophila*, we used a genetically encoded DEVDase probe consisting of a transmembrane mouse CD8 (mCD8) protein and a yellow fluorescent protein (Venus) linked by the caspase-3-cleavage sequence derived from human poly ADP ribose polymerase (PARP) [13] (Figure 1A). The activated form of DEVDase cleaves this probe, known as mCD8::PARP::Venus, into two fragments. Moreover, an antibody against cleaved PARP (anti-cPARP Ab) can specifically detect one of these two fragments; the immunohistochemical cPARP signal thus generated reflects levels of activated DEVDase.

**Figure 2:**
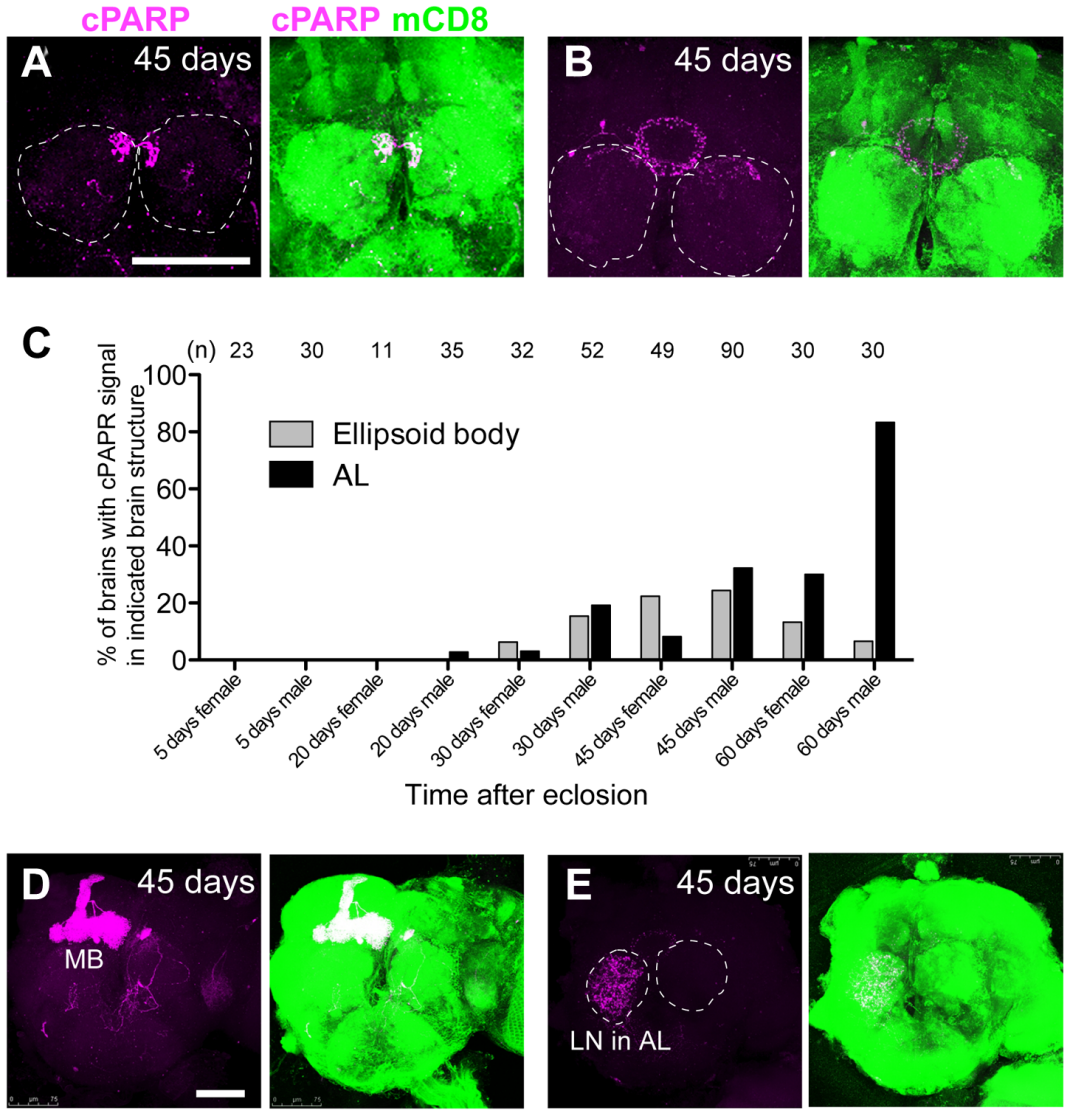
Stereotyped DEVDase activation in the AL and EB structures of aged *Drosophila* brains. (A, B, D, E) Representative aged brains (45-days-old) bearing cPARP signals (DEVDase activity) in the dorso-medial side of the AL (A), the EB structure (B), the mushroom body (MB) (D) and the local interneuron (LN) of the AL (E). mCD8::PARP::Venus was expressed in most postmitotic neurons (*elav-Gal4;;UAS-mCD8::PARP::Venus*). Circles of broken lines are ALs. cPARP signal and mCD8::PARP::Venus expression are shown in magenta and green, respectively. Scale bar: 75 µm. (C) Percentages of brains with cPAPR signal in the EB and AL at each time point are shown. “n” indicates the number of brains examined. Genotypes: (A–E) *elav-Gal4;;UAS-mCD8::PARP::Venus.*

We expressed mCD8::PARP::Venus in postmitotic adult neurons marked by *elav-Gal4* and found that the brains of very young (0–1 day old) and very old (30–45 days old) flies exhibited higher cPARP signaling frequency than other age groups (Figure 1B). In young adult brains, cPARP signals were primarily detected in the subesophageal ganglion (SOG) and in the midline region; however, the intensity of these signals varied in the SOG of individual brains (Figure 1C and 1D). The cPARP brain pattern was similar between males and females, although cPARP appeared more frequently in males than in females (Figure 1B, 1F and 2C). These results are consistent with previous findings obtained using the terminal deoxynucleotidyl transferase dUTP nick end labeling (TUNEL) assay and an antibody aimed to detect active forms of caspase-3 [26]. In contrast, brains of aged flies tended to exhibit cPARP in the dorso-medial corner of the AL and in the ellipsoid body (Figure 2A–2C). Other neuronal processes showed cPARP signals in the aged brain, but the labeling appeared to be random (Figure 2D and 2E; some data not shown). Importantly, we determined that the cPARP pattern in the AL was highly stereotyped in aged flies of both sexes (32.2% of male brains and 8.2% of female brains, at 45 days post-eclosion); hence, we focused on the AL neural circuit.

### DEVDase was activated in select olfactory neurons in an age-dependent manner

**Figure 3:**
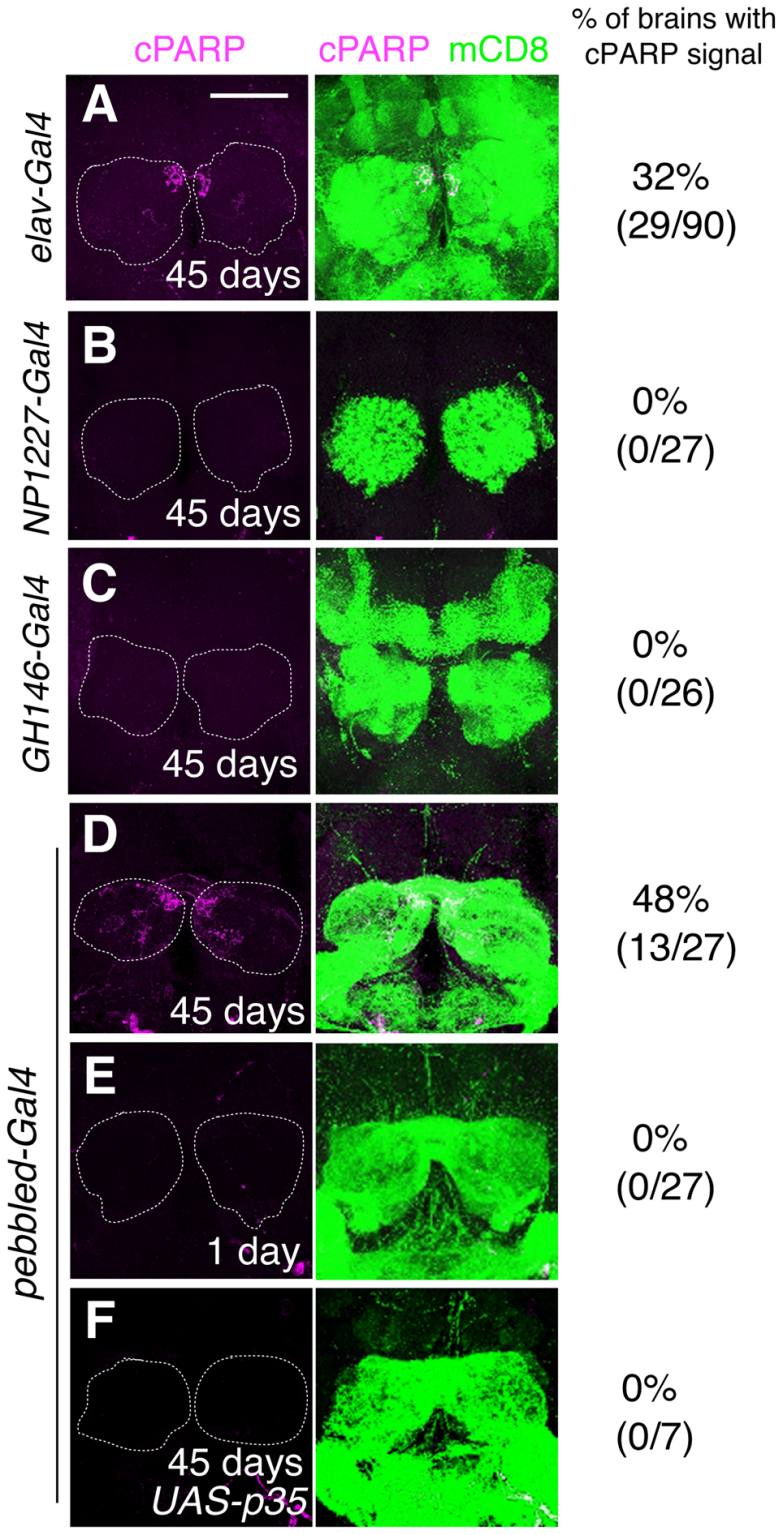
DEVDase is activated in the ORNs of aged *Drosophila* brains. (A–F) mCD8::PARP::Venus was expressed by *elav-Gal4* (A: all postmitotic neurons), *NP1227-Gal4* (B: GABAergic LNs), *GH146-Gal4* (C: two-thirds of the projection neurons), and *pebbled-Gal4* (D–F: all olfactory receptor neurons [ORNs]). ORNs of brains dissected from 45-day-old but not from 1-day-old flies had cPARP signals (D, E), which were inhibited by the expression of *p35*, a caspase inhibitor (F). cPARP signals and mCD8 staining are shown in magenta and green, respectively. Scale bar: 75 µm. Genotypes: (A) *elav-Gal4;;UAS-mCD8::PARP::Venus*. (B) *NP1227-Gal4;UAS-mCD8::PARP::Venus*. (C) *GH146-Gal4;UAS-mCD8::PARP::Venus*. (D, E) *pebbled-Gal4;;UAS-mCD8::PARP::Venus*. (F) *pebbled-Gal4;UAS-p35/+;UAS-mCD8::PARP::Venus*.

**Figure 4:**
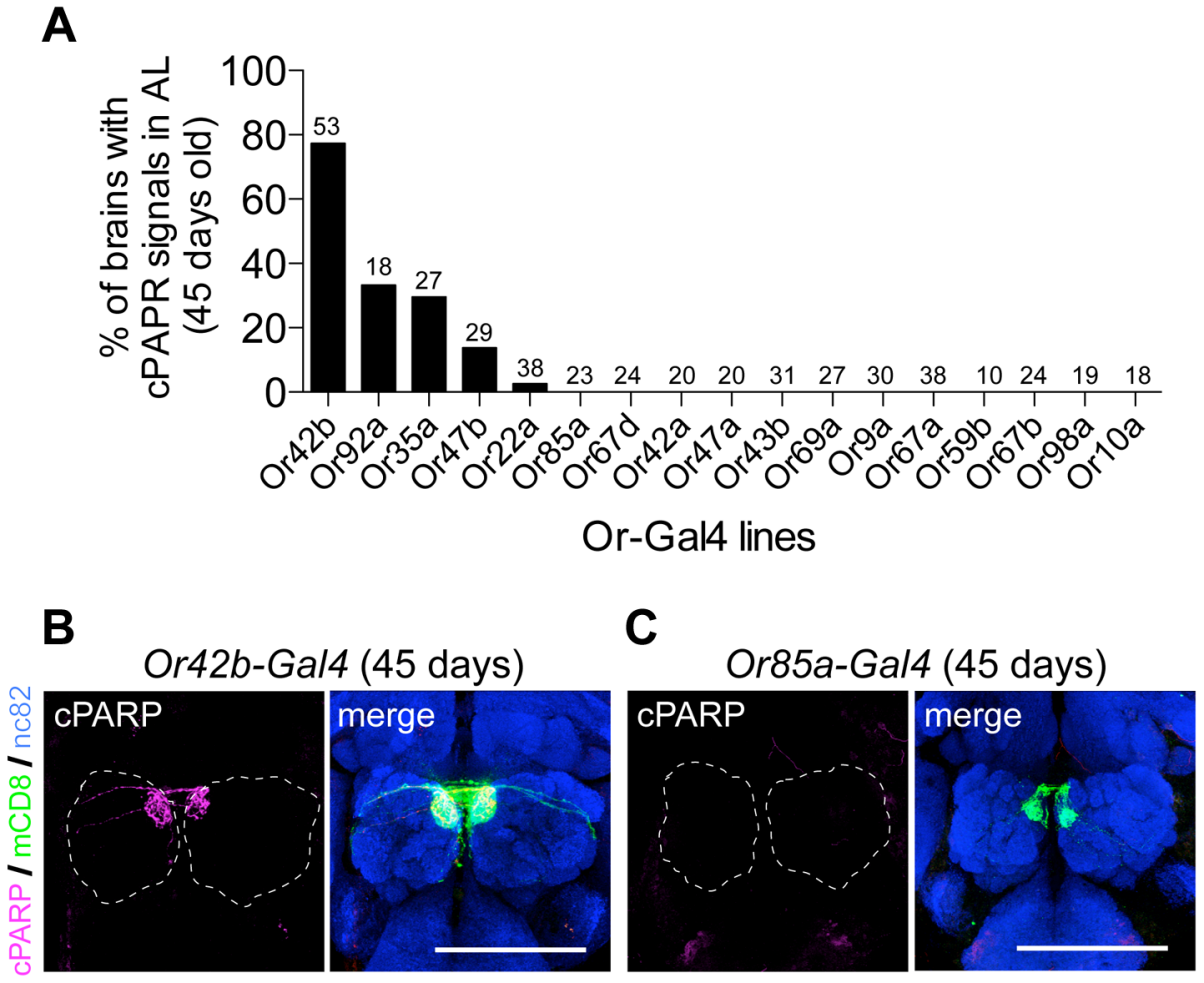
DEVDase is activated in a subset of ORNs in an age-dependent manner. (A) Percentages of aged brains (45-days-old) with cPAPR signals are shown. mCD8::PARP::Venus expression by various *Or-Gal4* drivers revealed that DEVDase is activated in a subset of ORNs in an age-dependent manner. Number on each column indicates the number of brains examined. (B, C) Representative images of aged fly brains expressing mCD8::PARP::Venus by *Or42b-Gal4* (B) or *Or85a-Gal4* (C). Note that cPARP signal was frequently observed in the axons of Or42b neurons (B) while it was rare in axons of Or85a neurons (C). cPARP signal, mCD8::PARP::Venus expression, and nc82 staining are shown in magenta, green, and blue, respectively. Broken lines indicate outlines of ALs. Scale bar: 75 µm. Genotypes: (A) Or42b: *Or42b-Gal4/+;UAS-mCD8::PARP::Venus/+*. Or92a: *Or92b-Gal4/UAS-mCD8::PARP::Venus;UAS-mCD8::PARP::Venus/+*. Or35a: *Or35a-Gal4/+;UAS-mCD8::PARP::Venus/+*. Or47b: *Or47b-Gal4/+;UAS-mCD8::PARP::Venus/+*. Or22a: *Or22a-Gal4/+;UAS-mCD8::PARP::Venus/+*. Or85a: *Or85a-Gal4/+;UAS-mCD8::PARP::Venus/+*. Or67d: *Or67d-Gal4, yw/+;;UAS-mCD8::PARP::Venus/+*. Or42a: *Or42a-Gal4/+;UAS-mCD8::PARP::Venus/+*. Or47a: *Or47aGal4/+;UAS-mCD8::PARP::Venus/TM2 or TM6B*. Or43b: *Or43b-Gal4/+;UAS-mCD8::PARP::Venus/+*. Or69a: *Or69a-Gal4/+;UAS-mCD8::PARP::Venus/+*. Or9a: *Or9a-Gal4/+;UAS-mCD8::PARP::Venus/+*. Or67a: *Or67aGal4/+;UAS-mCD8::PARP::Venus/+*. Or59b: *Or59b-Gal4, w/+;UAS-mCD8::PARP::Venus/+;UAS-mCD8::PARP::Venus/+*. Or67b: *Or67b-Gal4/UAS-mCD8::PARP::Venus*. Or98a: *Or98aGal4/+;UAS-mCD8::PARP::Venus/+*. Or10a: *Or10a-Gal4/+;UAS-mCD8::PARP::Venus/TM2 or TM6B*. (B) *Or42b-Gal4/+;UAS-mCD8::PARP::Venus/+.* (C) *Or85a-Gal4/+;UAS-mCD8::PARP::Venus/+*.

The AL is the first olfactory center in the *Drosophila* brain that consists of ∼50 glomeruli, which are ball-shaped synaptic structures that receive axons of ORNs and dendrites of projection neurons (PNs) and are interconnected by local interneurons (LNs) [22]. To identify the neuronal subtypes with DEVDase activity, we expressed mCD8::PARP::Venus using *pebbled-Gal4* (all ORNs), *NP1227-Gal4* (GABAergic LNs), and *GH146-Gal4* (two-thirds of the PNs). Only *pebbled-Gal4* generated reproducible cPARP signals in aged fly brains, which were suppressed by *p35* (Figure 3). Further, we used 17 *Or-Gal4* drivers to express mCD8::PARP::Venus in each ORN subtype. Surprisingly, we found that DEVDase was frequently activated in the axons of the Or42b, Or92a, and Or35a neurons but rarely in the other classes of ORNs that we tested (Figure 4A–4C). Or47b neurons also showed DEVDase activation, but cPARP signal intensity in these cells was very low. These data indicate that DEVDase activation is age-dependent in specific subsets of ORNs.

### DEVDase activity in Or42b and Or92a neurons causes age-related cell death

**Figure 5:**
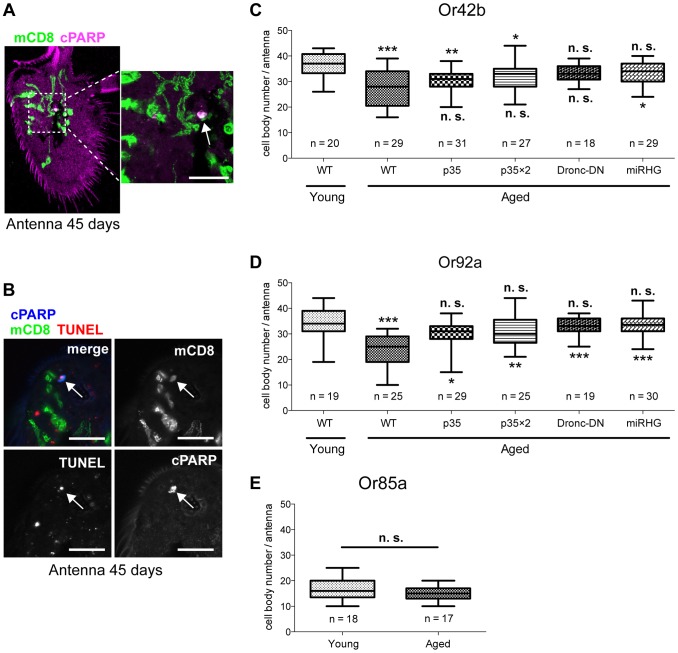
Selected Or42b and Or92a neurons die in an age-dependent, apoptotic manner. (A) cPARP signal was detected in cell bodies of ORN-expressing mCD8::PARP::Venus. Representative image (the right panel is a magnified view of the region outlined in the left panel) of an antennal-segment cryosection from an aged fly (45-days-old). cPARP signal and mCD8::PARP::Venus expression are shown in magenta and green, respectively. Arrow in the right panel indicates a cPARP-positive cell body of an Or42b neuron. The complete structure of the third antennal segment was traceable by autofluorescence in the magenta channel. Scale bar: 10 µm. (B) Representative image of the TUNEL assay with cPARP staining. TUNEL, cPARP, and mCD8::PARP::Venus expression (mCD8) are shown in red, blue, and green, respectively. The arrow indicates the cell body of a Or42b neuron positive for both TUNEL and cPARP signal. Scale bar: 10 µm. (C–E) Cell body numbers for each antenna are shown. Data were tested by Kruskal-Wallis test, followed by Dunn's multiple comparison test (C, D) or by an unpaired t-test (E). *p<0.05, **p<0.01, ***p<0.001, n.s.: no significance. Statistical results against young wild-type (WT) or aged WT are shown above or below each box plot, respectively (C, D). Genotypes: (A, B) *Or42b-Gal4/+;UAS-mCD8::PARP::Venus/+*, (C, D) WT: *w;OrX-Gal4/+;UAS-H2B::ECFP/+*, p35: *w;OrX-Gal4/UAS-p35;UAS-H2B::ECFP/+*, p35X2: *w;OrX-Gal4/UAS-p35;UAS-H2B::ECFP/UAS-p35*, Dronc-DN: *w;OrX-Gal4/+;UAS-H2B::ECFP/UAS-Dronc-DN*, miRHG: *w*;*OrX-Gal4/UAS-miRHG;UAS-H2B::ECFP/+*, X = *42b* (C), *92a* (D). (E) *w;Or85a-Gal4/+;UAS-H2B::ECFP/+*.

Strong activation of DEVDase in a cell body typically leads to apoptosis [5]. To determine whether DEVDase activation in Or42b and Or92a neurons caused apoptosis, we examined the activation state of DEVDase in ORN cell bodies located in the third segment of the fly antenna. In aged flies, among mCD8::PARP::Venus-positive Or42b and Or92a neurons, we found that a small fraction of neurons were positive for cPARP (Figure 5A): 6.7% of Or42b (n = 60 cells) and 3.1% of Or92a (n = 65 cells). On the other hand, none of the ORN cell bodies showed cPARP signals in young flies (0% Or42b neurons, n = 71 cells; 0 of Or92a neurons, n = 81 cells). In addition, some Or42b neurons are positive for both TUNEL and cPARP signal (Figure 5B). To further examine death of ORNs of aged flies, we expressed a nuclear-localized enhanced cyan fluorescent protein (ECFP) (Histone H2B::ECFP) in each type of ORN and found that there was a significant decrease in Or42b and Or92a neurons during aging (Figure 5C and 5D), while the number of Or85a neurons remained constant (Figure 5E). The expression of several apoptosis inhibitors (p35; the dominant-negative form of *Drosophila* caspase-9 Dronc, Dronc-DN [27]; and microRNA for *reaper*, *hid*, and *grim*, miRHG [28]) effectively reversed the trend of an age-dependent decrease in Or42b and Or92a neurons (Figure 5C and 5D). In contrast, the expression of these apoptosis inhibitors did not affect neuron numbers in young flies (Figure S1). These data suggest that Or42b and Or92a neurons die, at least in part, by caspase-mediated cell death in an age-dependent manner.

### Age-related impairments of attraction behavior and neural transmission are restored by caspase inhibition in corresponding ORNs

**Figure 6:**
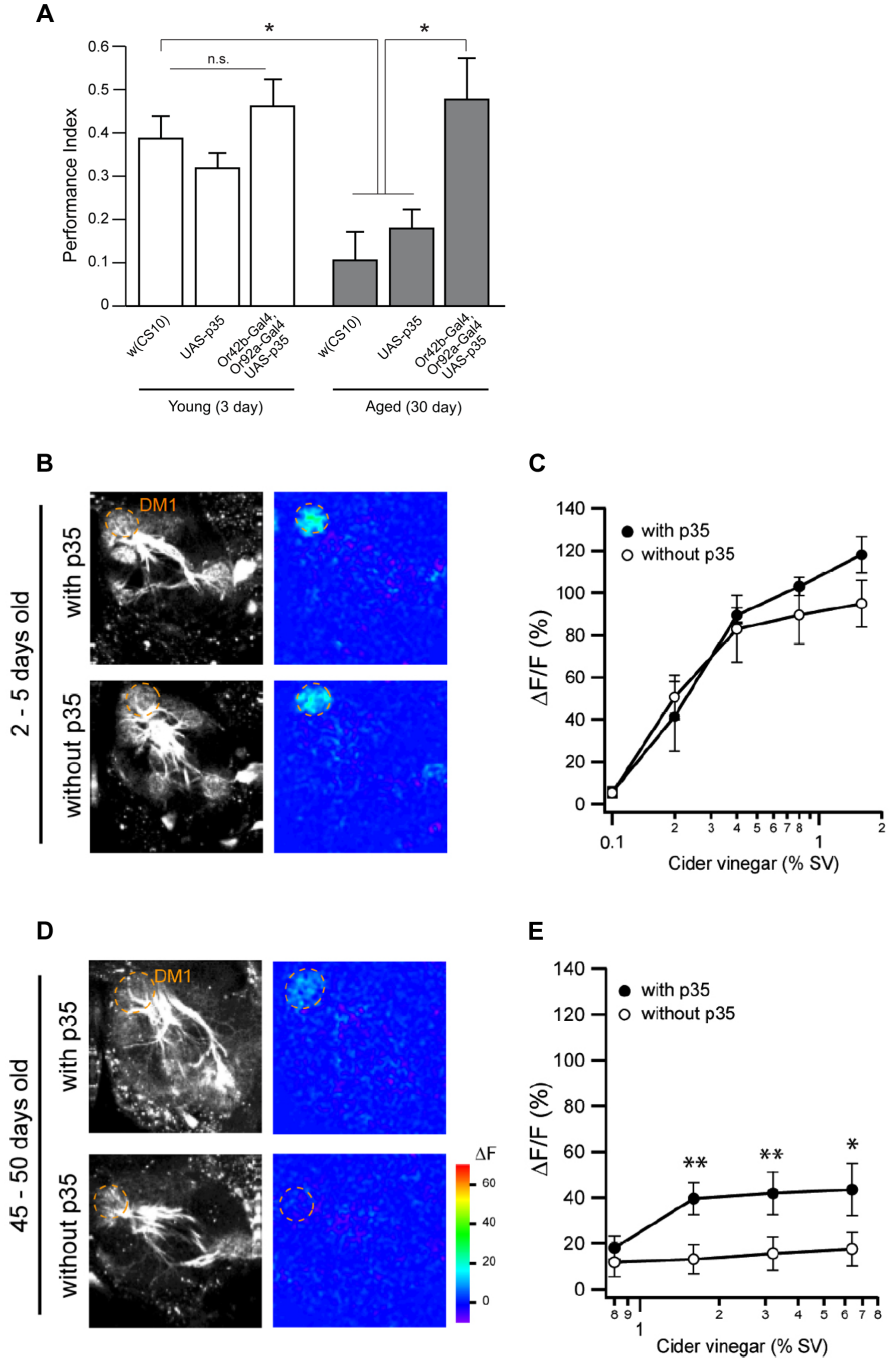
Age-related impairments of attraction behavior and neural transmission are restored by caspase inhibition in corresponding ORNs. (A) Performance index of flies bearing the *Or42b-Gal4*, *Or92a-Gal4*, and *UAS-p35* transgenes in response to apple cider vinegar vapor in 3 or 30-day-old flies. The performance index (P.I.) to apple cider vinegar between groups was not significantly different at 3 days of age. The P.I. in *w(CS10)* was significantly lower in 30-day-old flies than in 3-day-old flies. The 30-day-old flies carrying the *Or42b-Gal4* and *Or92a-Gal4* drivers and the *UAS-p35* transgene had an enhanced P.I. compared to flies of *w(CS10)* or flies bearing only the *UAS-p35* transgene at 30 days of age. Analysis of variance (ANOVA) followed by Tukey's test was performed on P.I. values from flies of 3 or 30-day-old flies. n ≧ 7 for all groups. *p<0.05. All error bars indicate the SEM. (B–E) Two-photon imaging of PN calcium response in flies with or without expression of *p35* in Or42b neurons. GH146-positive PNs express the calcium sensor GCaMP. Response to apple cider vinegar stimulation in 2–5-day-old flies (B, C) and 45–50-day-old flies (D, E). Grayscale images show the structure of the AL. Pseudo-colored images show the response to apple cider vinegar at 0.8% SV (saturated vapor pressure) (B) and 3.2% SV (D). DM1 peak fluorescence change ΔF/F is plotted against odor concentration for 2–5-day-old flies (C) and 45–50-day-old flies (E). n = 6–10. Error bars show SEM. *p<0.05, **p<0.01 (Wilcoxon signed-rank test). Only male flies were used. All flies contain *GH146-LexA* and *LexAop-GCaMP1.3*-*ires*-*GCaMP1.3*. Flies with *p35* expression also have *Or42b-Gal4* and *UAS-p35*.

Since the above-mentioned ORN subtypes die with DEVDase activation during aging, we hypothesized that odor-evoked behavior through the Or42b and Or92a neurons would be impaired with age. To test this possibility, we measured innate attraction behavior to apple cider vinegar in young and aged flies. Apple cider vinegar excites six glomeruli including DM1 and VA2, which are innervated by the axons of Or42b and Or92a, respectively [25]. We found that attraction to apple cider vinegar was significantly decreased in aged flies, and that this effect was reversed by p35 expression in Or42b and Or92a neurons (Figure 6A). These results clearly indicate that DEVDase activation in Or42b and Or92a neurons is the main cause of age-related impairments to innate attraction behavior.

Lastly, we wanted to investigate age-dependent change of glomerular sensitivity by using two-photon microscopy imaging with a genetically encoded calcium sensor, GCaMP [29]. We measure the sensitivity of a given glomerulus by monitoring its output projection neurons. Specifically, we image the dendritic calcium levels of PNs innervating each glomerulus, we applied apple cider vinegar to the flies bearing *GH146-LexA* and *LexAop-GCaMP1.3*-*ires*-*GCaMP1.3*. We found that, in young fly brains, the DM1 glomerulus was robustly activated in response to apple cider vinegar in a concentration-dependent manner (Figure 6B and 6C). In contrast, the DM1 glomerulus was only weakly activated even at high concentrations of apple cider vinegar in aged flies. Moreover, the expression of p35 in Or42b neurons increased sensitivity of DM1 to vinegar (Figure 6D and 6E). These results are consistent with our observations on attraction behavior (Figure 6A), and indicate that the olfactory response of the DM1 glomerulus is impaired during aging due to DEVDase activation in Or42b neurons.

## Discussion

In the current study, we demonstrate that normal aging increases caspase activity, leading to age-related cell death, reduced olfactory sensitivity, and impaired innate attraction behavior. Since caspase-3 activity appears to contribute to synaptic LTD in the rat hippocampus [17], [18] and to early synaptic dysfunction in mouse models of Alzheimer's disease [20], further studies of age-related increases in caspase activity and its role in *Drosophila* neuronal excitability and cell death are warranted. Our discovery that specific types of ORNs show DEVDase activation and cell death is the first example of age-related, stereotyped cell death of neurons within a specific network. The activation of the DM1 and VA2 glomeruli, which are innervated by Or42b and Or92a neurons, respectively, is essential for innate food attraction behavior [25]. Thus, our observations may help to explain age-related changes in innate animal behavior.

In addition to the olfactory system, we found stereotyped caspase activation in the ellipsoid body, the brain region involved in olfactory memory consolidation [23] and visual place memory [24]. This observation could imply the possible contribution of caspase activation to age-related memory impairment (AMI). Like other animals, aged flies exhibit AMI, which corresponds to an increase of cAMP-dependent protein kinase (PKA) in the mushroom body but not in the ellipsoid body [30]. Because caspase is required in synaptic LTD [17], it might be interesting to investigate the possible implication of caspase activation in the ellipsoid body for olfactory or visual memory and whether its role is apoptotic or non-apoptotic.

The results of our current study reveal an interesting phenomenon in that age-related caspase activation only occurred in select ORNs. One possible explanation for this is the continuous activation of Or42b and Or92a neurons by food odors. As previously discussed, Or42b and Or92a respond to odors that flies recognize as food, such as apple cider vinegar [25]. Under regular experimental conditions, flies are cultured in a food-containing vial leading to continuous activation of Or42b and Or92a for the duration of a fly's lifespan. To test whether this continuous ORN activation was responsible for the eventual age-related caspase activation in these neurons, we aged flies in a vial containing yeast paste and examined cPARP signal in Or42b neurons. Interestingly, we found that the age of onset and strength of caspase activation in Or42b neurons was similar to what we found in flies that had been cultured in normal food (data not shown), suggesting that a continuous food odor is not solely responsible for inducing age-related caspase activation. As for the involvement of neural activity in the maintenance of ORN axons [31], further studies of culturing conditions containing restricted odors and the genetic manipulation of ORN neural activity would help to clarify these issues.

In addition to neuronal activity, aging itself might produce ORNs with differential sensitivity to neuronal excitability or toxicity. This idea is supported by a report from Tonoki et al. (2011), showing that forced expression of a truncated form of human Machado-Joseph disease protein with an expanded polyglutamine domain in the adult *Drosophila* eye at 20–24 days after eclosion causes more severe neurodegeneration than expression at 0–4 days of age [32]. These observations suggest that neuronal identity, including sensitivity to a toxic factor generated by age-related neuronal excitability, may be continuously changing over the course of a fly's lifecycle.

Differential expression of the effector caspases, drICE and Dcp-1, may determine the spatiotemporal specificity of caspase activation. Previous studies have suggested that expression levels of these caspases reflect the apoptotic potential of cells, and that drICE is more effective than Dcp-1 to induce apoptosis [33]. Interestingly, it has been shown that activation of drICE and Dcp-1 can only be detected in degenerating dendrites but not in the cell body of *Drosophila* C4da neurons [34]. We also previously reported a similar phenomenon in mice where caspase-3 could be detected in the developing axons of olfactory sensory neurons but not in the cell body [19]. In the current study, we found that caspases were activated in both the axon and cell body of a subset of Or42b neurons that eventually go on to die in an apoptotic manner, while the subset that did not show elevated levels of caspases went on to survive. Therefore, we expect that both drICE and Dcp-1 were likely activated in dying Or42b neurons, while either drICE or Dcp-1 was activated in the degenerating axons of surviving Or42b neurons.

It has been reported that the Or42b and Or92a genes are the most conserved in the drosophilid olfactory subgenome and seem to be utilized to detect odors from wild lily (Solomon's lily) in other drosophilid species [35]. Thus, Or42b and Or92a seem to possess the most fundamental function among the ∼50 types of olfactory neurons. Moreover, this suggests that caspase activation in these neurons might have a greater impact on animal behavior. Therefore, we believe that this would be an ideal experimental paradigm to investigate age-dependent changes of innate behaviors.

Lastly, our current findings suggest that while it is clear that caspase activation plays a crucial apoptotic role in the adult olfactory circuit, caspase activation may also have non-apoptotic functions. This is in light of that fact that while we were only able to detect a few TUNEL-positive cells among cPARP-positive ORNs, we noted a significant reduction in odor-evoked neural activity in the DM1 glomerulus of aged flies. Richard et al. recently identified an age-dependent disruption of a specific synaptic layer in the mouse olfactory bulb without any detectable neuronal loss [36]. In addition, it has been shown that caspase-9 is activated in aged olfactory bulb neurons without affecting the number of these cells [37]. These observations, together with our study, prompt questions concerning the ecological and pathological significance of caspase activation, or synaptic dysfunction, in specific groups of neurons or synapses of the adult olfactory circuit. Investigating the relationship between age-related alterations in neural circuits may provide clues to understanding the neural basis of impaired sensory and cognitive performance during normal aging and senile dementia.

## Materials and Methods

### Fly stock and culturing condition

The following transgenic lines were used: *elav-Gal4*, *Or-Gal4*, (Bloomington Stock Center), *NP1227-Gal4* (Kyoto *Drosophila* Stock Center), *UAS-mCD8::PARP::Venus* [13], *pebbled-Gal4* [38], *GH146-Gal4* [39], *UAS-miRHG* [28], *UAS-reaper* [40], *UAS-p35* (a gift from Bruce Hay), *UAS-Dronc-DN* [27], *UAS-mCD8::GFP* [41], *UAS-H2B::ECFP* [42], *LexAop-GCaMP1.3-ires-GCaMP1.3* [25], and *GH146-LexA* [43]. All flies were maintained in a 25°C incubator and transferred to vials with fresh food every 3 to 4 days.

### Immunohistochemistry

Immunohistochemistry of the *Drosophila* adult brain was performed as previously described [44]. To stain ORN cell bodies, we dissected antennae from flies, fixed them in 4% (vol/vol) paraformaldehyde/0.3% (vol/vol) phosphate-buffered saline with Triton X-100 (PBT) at room temperature (R.T.) for 30 min, mounted them in OCT, and cut 14 µm-thick sections on a cryostat. Slides were then re-fixed with 4% (vol/vol) paraformaldehyde/0.3% (vol/vol) PBT at R.T. for 30 min, washed with 0.3% (vol/vol) PBT, and labeled using standard techniques. Antibodies used include rat anti-mouse CD8 antibody (1∶100, MCD0800, Invitrogen), rabbit anti-cleaved PARP (Asp214) antibody (1∶100, #9541, Lot.7, Cell Signaling), rabbit anti-cleaved PARP antibody [Y34] (1∶100, ab32561, Abcam), nc82 mouse monoclonal antibody (1∶40, Developmental Studies Hybridoma Bank), anti-rat Alexa488 (1∶250), anti-rabbit Cy3 (1∶1000), and anti-mouse Cy5 (1∶1000) (Jackson Laboratory). Confocal images were captured using a Leica SP5 confocal microscope.

### TUNEL assay

TUNEL assay of antennal cryosections was performed using an *In Situ* Cell Death Detection Kit, TMR red (Roche). Tissues were mounted in SlowFade Gold Antifade Reagent with DAPI (Life Technologies).

### Behavioral assay

Cantonized *w^1118^* [*w(CS10)*] flies were used as behavioral controls in our experiments. The flies used for behavioral assays were out-crossed to the *w(CS10)* background. All fly stocks were maintained at 25°C and 70% relative humidity under a 12/12 h light-dark cycle. For behavioral studies, about 50 male flies were placed into food vials and transferred to fresh food vials every 3 or 4 days until the age for behavioral assay was reached. Behavioral assays were performed under dim red light at 25°C and 70% relative humidity. Attraction to apple cider vinegar was measured for 3 or 30-day-old flies. Briefly, flies of each type were loaded into a T maze in which they could make a choice between two arms. Flies were allowed 2 min to choose between an odor of apple cider vinegar or air. Apple cider vinegar was diluted in water to 0.3% (v/v), which elicits robust attraction behavior in 3-day-old flies. The performance index (P.I.) was defined as the ratio of the difference in number of flies that chose the air laced with or without apple cider vinegar odor to the total number of flies that chose either side.

### Two-photon imaging with G-CaMP

Calcium imaging was performed as described [25], [29], [45]. For odor stimulation experiments, a constant airflow of 1 L/min was applied to the antennae via a tube of 12 mm diameter. Odor onset was controlled by mixing a defined percentage of carrier air redirected through odor bottles (presented as percent saturated vapor pressure, or %SV) as previously described [45].

### Enumerating ORN cell numbers in the antennae

Third segments of the antennae were dissected in phosphate-buffered saline (PBS) and mounted with FocusClear mounting solution (Cedarlane Laboratories). All cell images (H2B::ECFP) were taken live by a Leica SP5 confocal microscope within 15 min. Cell numbers were manually counted with ImageJ software and statistical analyses were performed using Stastical Package for the Social Sciences (SPSS 16.0) (IBM) and Prism software (GraphPad).

## Supporting Information

Figure S1 Expression of apoptosis inhibitors does not affect the ORN cell number in young flies. Cell body numbers in each antenna of young flies are shown. Data were subjected to an Unpaired t-test, Unpaired t-test with Welch's correction, or Mann-Whitney U-test. n.s.: no significance. Genotypes used in these analyses: WT: *w; OrX-Gal4/+; UAS-H2B::ECFP/+*, p35: *w;OrX-Gal4/UAS-p35;UAS-H2B::ECFP/+*, p35X2: *w; OrX-Gal4/UAS-p35;UAS-H2B::ECFP/UAS-p35*, Dronc-DN: *w;OrX-Gal4/+; UAS-H2B::ECFP/UAS-Dronc-DN*, miRHG: w*;OrX-Gal4/UAS-miRHG;UAS-H2B::ECFP/+*, X = *42b* (A), *92a* (B).
(PDF)
