# Supplementary figures and images for: Caspase Inhibition in Select Olfactory Neurons Restores Innate Attraction Behavior in Aged Drosophila
Source: PLoS Genet. 2014 Jun 26;10(6):e1004437. doi: 10.1371/journal.pgen.1004437 (PMC4072539; doi:10.1371/journal.pgen.1004437)

Figure S1

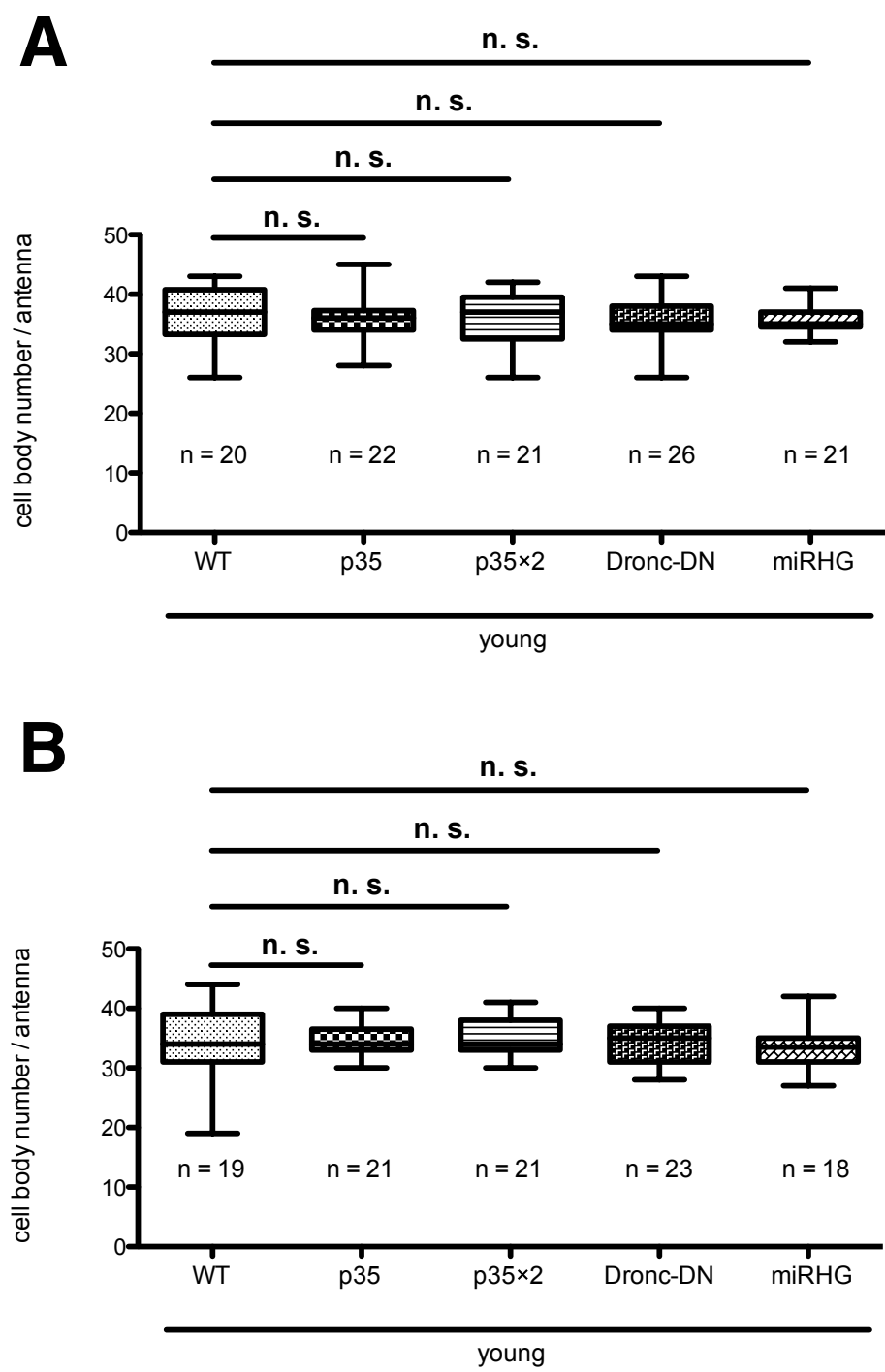

Supplement: Figure S1 — Expression of apoptosis inhibitors does not affect the ORN cell number in young flies. Cell body numbers in each antenna of young flies are shown. Data were subjected to an Unpaired t-test, Unpaired t-test with Welch's correction, or Mann-Whitney U-test. n.s.: no significance. Genotypes used in these analyses: WT: w; OrX-Gal4/+; UAS-H2B::ECFP/+, p35: w;OrX-Gal4/UAS-p35;UAS-H2B::ECFP/+, p35X2: w; OrX-Gal4/UAS-p35;UAS-H2B::ECFP/UAS-p35, Dronc-DN: w;OrX-Gal4/+; UAS-H2B::ECFP/UAS-Dronc-DN, miRHG: w;OrX-Gal4/UAS-miRHG;UAS-H2B::ECFP/+, X = 42b (A), 92a (B). (PDF) [file pgen.1004437.s001.pdf]
